# Piano Wire Clamps

**Published:** 1902-03

**Authors:** Herbert L. Wheeler

**Affiliations:** 12 West Forty-sixth Street; New York


### Domestic Correspondence.

### PIANO WIRE CLAMPS.

New York, December 31, 1901.

To the Editor:

Sir,—I desire to send to you for publication an article, or rather item, concerning Piano Wire Clamps. These were first suggested by Dr. H. F. Harvey, of Cleveland, Ohio, in a paper read before the Ohio State Dental Society, and published in the *Dental Cosmos* for March, 1900. The profession owes Dr. Harvey a debt of gratitude for originating this useful appliance and then giving it to them without covering it with a patent, as is too common when an apparently new contrivance is introduced. It seems to me that this clamp has not received an amount of attention at all proportionate to its value.

If any one will take the requisite time to learn to make these clamps (the process is described in Dr. Harvey's paper), they will certainly be amply repaid. The ease with which they can be adjusted to teeth upon which any ordinary clamp will not stay while it is being put on, and the saving in time to the dentist and of annoyance to the patient are factors that make this instrument almost invaluable. Dr. F. L. Bogue has made one suggestion as to the method of making them that facilitates the operation greatly. Where Dr. Harvey uses a matrix, filed from an old instrument, to hold the two parts of the clamp together, Dr. Bogue simply winds tightly with ordinary brass or steel wire and then flows the soft solder over this.

In the office of Dr. J. Morgan Howe we take an impression in very difficult cases and make up a clamp especially for that tooth. In cases of partially erupted or conical shaped molars we have been able to hold the dam on with these clamps where no ordinary clamp would work.

Very truly,

Herbert L. Wheeler.

12 West Forty-sixth Street.

